# Integrative transcriptomics analysis reveals the metabolic regulatory functions of lncRNA in the livers of yak at different age stages

**DOI:** 10.1371/journal.pone.0333944

**Published:** 2025-10-08

**Authors:** Hongsen Fu, Wei Xia, Fang Fu, Jun Yi, Wei Wang, Yong Wei, Li Wang

**Affiliations:** 1 Key Laboratory of Qinghai-Tibetan Plateau Animal Genetic Resource Reservation and Utilization, Ministry of Education and Sichuan province, Southwest Minzu University, Chengdu, China; 2 College of Animal Science and Technology, Heibei Agricultural University, Baoding, China; 3 Sichuan Animal Sciences Academy, Chengdu, China; Nuclear Science and Technology Research Institute, IRAN, ISLAMIC REPUBLIC OF

## Abstract

Exploring the regulatory role of long non-coding RNA (lncRNA) in plateau yak is crucial to understanding its metabolic network for adapting to extreme environments. By integrating transcriptomic sequencing and co-expression network analysis, the messenger RNA (mRNA) and lncRNA expression characteristics of yak liver at three growth and development stages were systematically analyzed. A total of 35,216 mRNAs and 10,073 lncRNAs were detected. Among the 288 differentially expressed lncRNAs, 88 lncRNAs related to metabolism were screened, and their potential functions in lipid metabolism, collagen remodeling, and protein transport were predicted. The age-dependent expression patterns of some lncRNAs were verified through qRT-PCR (quantitative real-time reverse transcription polymerase chain reaction) experiments, which initially revealed the status and role of lncRNAs in metabolic regulation in yak liver. This study provides new insights into the molecular mechanisms underlying metabolic adaptation in high-altitude species such as yak, and establishes a methodological framework for the screening and identification of functional lncRNAs in non-model organisms.

## Introduction

Long non-coding RNAs (lncRNAs) were once considered transcriptional noise, but have now been proven to be key regulators of gene expression and play an important role in biology [1]. Although significant progress has been made in recent years in the study of the regulation of individual metabolic pathways, there are still many unknown areas in the complex regulatory network of metabolic physiology. For metabolic diseases such as diabetes and obesity, it is still difficult to develop effective treatment strategies [2–4]. Emerging evidence indicates that the lncRNA LINK-A exerts indirect regulatory effects on adipose tissue metabolism through inflammatory pathway activation. The underlying mechanism involves a self-reinforcing feedback loop comprising the LINK-A/HB-EGF/HIF1α axis, which drives pathological remodeling of the adipose tissue microenvironment and impairs adaptive thermogenic capacity. This molecular cascade ultimately manifests as metabolic dysfunction characterized by obesity development and insulin resistance progression [5]. Nearly two-thirds of transcripts are non-coding RNAs, mainly originating from regions previously considered junk genes [6]. Among the non-coding RNAs identified to date, lncRNAs are the longest and most difficult to understand. Their transcripts are 200 nucleotides (nt) or longer and do not have the potential to encode proteins. LncRNAs have been identified in all model organisms [7], and their number continues to grow [8–10]. Research shows that lncRNAs influence multiple aspects of cell function, including chromatin modification and transcriptional regulation, RNA stability, and translational regulation [11,12]. Evolutionary studies suggest that there may be more than 1,000 lncRNAs with conserved functions in mammals [13]. There are also many reports indicating that lncRNA plays a role in bovine embryonic development [14], skeletal muscle [15], and mammary glands [16]. In mice, in silico method had been used to identify functional lncRNAs in metabolic process [17]. Transcriptomic profiling integrated with competing endogenous RNA (ceRNA) network analysis in ducks identified a novel regulatory axis involving lncRNA-00742, miR-116 and CD74, which modulates vanadium-triggered mitochondrial apoptotic pathways [18]. However, for ruminants such as cattle or yaks, this field remains largely unexplored.

Metabolic processes are one of the most fundamental activities closely related to the basic biological and physiological activities of all animals [19]. As the most important metabolic organ, the liver continuously exchanges information with other organs through endocrine factors to maintain overall metabolic balance. It is also a complex digestive gland in ruminants (including yaks) and plays a key role in material metabolism. More importantly, the liver plays a crucial role during development [20–22]. There have been relatively few studies on lncRNAs in yak liver, and their role in liver development, metabolism, and disease onset is also poorly understood. Research by Dorland et al. indicates that there is significant metabolic adaptation during the transition period from the dry-off period to early lactation in dairy cows, with the liver playing a key role in this process [20].

Yaks (*Bos grunniens*) are distributed in the high-altitude regions of the Qinghai-Tibet Plateau in western China. This region has harsh environmental conditions, with low temperatures, dryness, large diurnal temperature variations, cold and long winters, low oxygen levels, and strong ultraviolet radiation. Yaks have adapted to thrive in these harsh environments, while most other domesticated animals struggle to survive there. For these reasons, yaks have become an important pillar of animal husbandry on the Qinghai-Tibet Plateau, providing local residents with meat, milk, and other important means of production and livelihood [23]. Therefore, exploring the role of lncRNA in metabolic regulation in yak is of great significance for the scientific feeding of yaks. This study selected newborn yak calves (1 day old), juvenile yak (15 months old), and adult yak (5 years old) as research materials. Through high-throughput RNA sequencing of lncRNA transcripts in the livers of yaks at three different developmental stages, a comprehensive workflow was established to identify lncRNAs that play a role in metabolic regulation. Network analysis of lncRNAs and mRNA was then used to predict the functions of lncRNAs.

## Materials and methods

### Animal

This study used a total of nine Maiwa yaks from the Yaks Technology Park in Hongyuan County of Sichuan Province in China, representing three different age groups (LD: 1 Day, LM: 15 Months, LY: 5 Years). The experimental animals were healthy and were kept under consistent feeding and management conditions. The weights of the yaks were 10.55 ~ 14.24 kg, 96.38 ~ 101.37 kg and 240.73 ~ 296.36 kg. All yaks were stunned using a captive bolt stunner (Cash 8000 model stunner, caliber 0.22 inches, using 4.5 grains of ammunition) to reduce the animal’s suffering prior to humane slaughter. Subsequently, bleeding was performed at the slaughterhouse through a transverse incision in the neck. The liver tissues were immediately removed and rapidly stored in liquid nitrogen until RNA extraction. Establish groups of yaks of different ages to analyze the transcriptome in liver tissues. All animal experiments were conducted according to the regulations for the Administration of Affairs Concerning Experimental Animals (Ministry of Science and Technology, China, revised in June 2004) and approved by the Institutional Animal Care and Use Committee in the Southwest Minzu University, Chengdu, China.

### RNA quantification and qualification

Total RNA was extracted from liver tissue using RNAiso Plus (TaKaRa, Japan). The degree of RNA degradation and contamination was monitored using a 1% agarose gel. The purity of RNA was determined using a NanoPhotometer^®^ spectrophotometer (IMPLEN, CA, USA). RNA concentration was measured using the Qubit^®^ RNA Assay Kit on a Qubit^®^ 2.0 Fluorometer (Life Technologies, CA, USA). The integrity of RNA was assessed using the RNA Nano 6000 Assay Kit on the Bioanalyzer 2100 system (Agilent Technologies, CA, USA). If the OD_260/280_ value of total liver tissue RNA was between 1.8 and 2.2, the quality test was considered satisfactory (Table 1) and subsequent analysis can proceed.

**Table 1:** Quality inspection results of RNA extracted samples.

Sample name	RNA concentration (ng/μL)	volume (μL)	Total quantity (μg)	OD_260/280_	OD_260/230_	Test conclusion
LD-1	1552	32	49.66	2.18	2.04	A
LD-2	1366	32	43.72	2.14	1.92	A
LD-3	1880	32	60.16	2.07	2.05	A
LM-1	1916	32	61.31	2.05	1.88	A
LM-2	1168	32	37.38	1.99	1.65	A
LM-3	1932	32	61.82	2.10	2.03	A
LY-1	1064	32	34.05	2.07	1.98	A
LY-2	1254	32	40.13	1.92	1.82	A
LY-3	1032	32	33.02	2.15	2.24	A

Note: The classification requirements for the test results are as follows: Rate A: the sample quality meets the requirements for library construction and sequencing, and the total quantity meets the requirements for library construction two or more times.

### Library preparation for high-throughput sequencing

Each sample was prepared using 3 μg of RNA as input material. Strand-specific libraries were constructed by removing rRNA, and sequencing libraries were generated using the NEBNext^®^ UltraTM RNA Library Prep Kit for Illumina^®^ (NEB, USA) [24]. Briefly, mRNA was purified from total RNA using magnetic beads modified with poly-T oligonucleotides. The first strand of complementary DNA (cDNA) was synthesized using random hexameric primers and Ribonuclease H (RNase H). Subsequently, the second strand of cDNA was synthesized using DNA polymerase I and RNase H. The purified double-stranded cDNA was end-repaired, poly-A tailed, and ligated with sequencing adapters. To select cDNA fragments with a length preference of 150–200 bp, library fragments were purified using the AMPure XP system (Beckman Coulter, Beverly, USA). Then, 3 μL of USER enzyme (NEB, USA) was used to degrade the second strand of cDNA containing uracil, and perform PCR (polymerase chain reaction) amplification to obtain the library. After the library was constructed, preliminary quantification was performed using a Qubit 2.0 fluorometer (Invitrogen, USA). PCR products were purified using the AMPure XP system, and library quality was assessed using the Agilent Bioanalyzer 2100 system. The effective concentration of the library was accurately determined using qRT-PCR (Bio-Rad, USA) to ensure library quality. After library preparation, sequencing was performed on the Illumina Hiseq platform, and 150 bp paired-end reads were generated.

### Transcriptome sequencing data analysis

Quality-controlled Clean Reads were aligned to the yak reference genome (BosGru_v2.0: http://ftp.ensembl.org/pub/release-97/fasta/bos_mutus/), using HISAT2 v2.0.5 software (http://ccb.jhu.edu/software/hisat2) for fast and accurate alignment to obtain aligned read sequences for subsequent analysis [25]. At the same time, the quality of the comparison results was evaluated. By analyzing the different regions and chromosome distribution of mapped reads in the reference genome, the alignment efficiency and mapping information of mapped reads in each sample can be obtained [26]. New transcripts mapped reads were assembled and quantified using StringTie (1.3.3b) software [27]. Sequencing depth and gene length were normalized using FPKM [28]. Differential expression analysis was performed using the DESeq2 R package (1.10.1). DESeq2 provides a set of statistical functions for performing differential expression analysis of numerical gene expression data based on a negative binomial distribution model. Genes with adjusted padj < 0.05 obtained through DESeq2 analysis were identified as differentially expressed genes [29].

### Function enrichment

ClusterProfiler (v3.4.4) software was used to perform Gene Ontology (GO) functional enrichment analysis and Kyoto Encyclopedia of Genes and Genomes (KEGG) pathway enrichment analysis on the differentially expressed gene sets to predict the biological processes and functions in which they may be involved, and to perform corresponding classification and statistics [30]. All lncRNAs were used for target gene prediction by predicting the target genes of lncRNAs based on the positional relationship (co-localization) and expression correlation (co-expression) between lncRNAs and protein-coding genes [31]. Subsequently, functional enrichment analysis (GO/KEGG) was performed on the target genes of differentially expressed lncRNAs to predict the main functions of lncRNAs.

### Real-time fluorescent quantitative PCR

Measurement of gene expression with qRT-PCR has been applied in our studies. Briefly, total RNA was extracted from liver tissue of each group using RNAiso Plus (TaKaRa, Japan). RNA was intact without degradation, of high quality and purity, and meeting the requirements for subsequent experiments. qRT-PCR was performed in a reaction system with a total volume of 10 µL, containing 5.2 µL of TB GreenTM Premix Ex TaqTM II (TaKaRa, Japan), 1 µL of cDNA, 0.8 µL of each primer (10 mM), and 2.2 µL of double-distilled water(ddH_₂_O). The reaction conditions were as follows: 95 °C for 3 min, followed by 39 cycles of 95 °C for 10 s, 52/53.4 °C *(*target gene**/*β-*actin**) for 20 s, 72 °C for 20 s, and a melting curve from 0.5 °C to 95 °C at a rate of 0.5 °C every 5 s. The experiment was performed using a real-time fluorescent quantitative polymerase chain reaction system (Bio-Rad, USA). For each sample, the cycle threshold (CT) values were obtained from three replicate experiments. *β-actin* mRNA was used as an internal reference. The primer sequences used for amplifying the target gene and internal reference gene are shown in Table 2. The relative expression levels of the target gene were analyzed using the 2^-ΔΔCT^ method.

**Table 2:** Primers sequence of the genes used for Real-Time fluorescence quantitative PCR.

	Name	ID	Primer sequences (5′–3′)	Tm/°C
LncRNA	TRPM8-OT1	TCONS_00166876	F: ACA GGC AAC GCA TTC AGA R: ACC GAG ATT CAG GGC TTC	52
	LINC4531	TCONS_00195143	F: AGT GCC TTC AGT TGT GCT R: ACT CAA AAG CAA GCG GTC	52
	NNAT-OT1	TCONS_00098793	F: ACA GGC AAC GCA TTC AGA R: ACC GAG ATT CAG GGC TTC	52
	DGKQ-OT1	TCONS_00031898	F: GTG TCT GTC GTC CGC AAA R: GCA ACT CCT GGC AAC TGT	52
	LINC3623	TCONS_00160902	F: GGT TGC CAC TCC CTT CTC R: CCT TCT TGG GCT TCC TTA	52
	LINC4526	TCONS_00194942	F: TCT TGC GAT GGA CTA TGC R: TCT TTG CCG AAT CAG GTG	52
	TCONS_00098792	TCONS_00098792	F: GCC GAA GCC CTG AAT CTC R: AGG GAG CAA AGG CAG CAA	58
mRNA	*β-actin*	AC_000162.1	F: CTT CGA GCA GGA GAT GGC R: CCG TGT TGG CGT AGA GGT	53.4
	*SPP2*	ENSBMUG00000025472	F: AAC AGT TAC CTG CTT GGC R: TTT ACT CTT GCT CTG GGC	52
	*FSTL1*	ENSBMUG00000026481	F: ACC AGG AGA ACA ACA AGC R: TTG AGG CAC TTG AGG AAC	52
	*NNAT*	ENSBMUG00000000490	F: CAC ACA CGC AGC ACG CAC A R: GCA GTC CCC AGC CGA GTG AAT	52
	*SLC26A1*	ENSBMUG00000024704	F: CAG TGA CCA TCC TGA CCT C R: AGC ACG TCA CAC AGG TTG	52
	*UGT2A2*	ENSBMUG00000018785	F: GTG AGA AAC ACT GGG CTG GAT A R: TGC CAT AAG GGT GGT GTC C	52
	*NOTUM*	ENSBMUG00000026356	F: TTC ATC CCC TAC TGC TCC R: CAG GTT GGA AGG AGG TCA	52
	*FASN*	ENSBMUG00000026205	F: TCC AGC ACA GCC AGA ACC R: CGG AGC AGA TGA ACC AGA	52
	*SEL1L*	ENSBMUG00000020767	F: TGG AAT GGC TTA CCT CTA R: TGT TTG TAA TCC CTC TTG AC	58
	*POLR2E*	ENSBMUG00000004215	F: TAC ATC TTG GAG CAG TTT CT R: GCC AGC AGT TCC GTT A	58
	*TUBB*	ENSBMUG00000007738	F: TGC CAA GTT CTG GGA GG R: CCAAGA TAG CAC GAG GG	58
	*GALT*	ENSBMUG00000018148	F: CTC ATT ATT ACC CTC CAC TC R: GCT GTC TCC CTG TCC TT	58
	*LGALS1*	ENSBMUG00000009672	F: GGG CAA AGA CGA CAA C R: GCA TAC CTC CAC GAC AC	58
	*LGALS3 BP*	ENSBMUG00000008997	F:AGT CCAATC CCA CCA TC R:AAC CTG CTG AAG AAC CCT	58
	*SLC22A7*	ENSBMUG00000012334	F:CAT CAT GTT TGC CAT CAC TC R:AAT CCC GTA TCA GGT AGC C	58
	*STR6L*	ENSBMUG00000002392	F:TCC TGT CAT CCA CCA T R:GCT TGT CAT CTG TCC C	58

### Data analysis

All data are presented as means ± SEM. Comparisons were made by 2-tailed Student’s t tests. Values of *P* < 0.05 were considered significant.

## Result

### Expression profiles of lncRNA and mRNA in yak liver at different age stages

**Fig 1:**
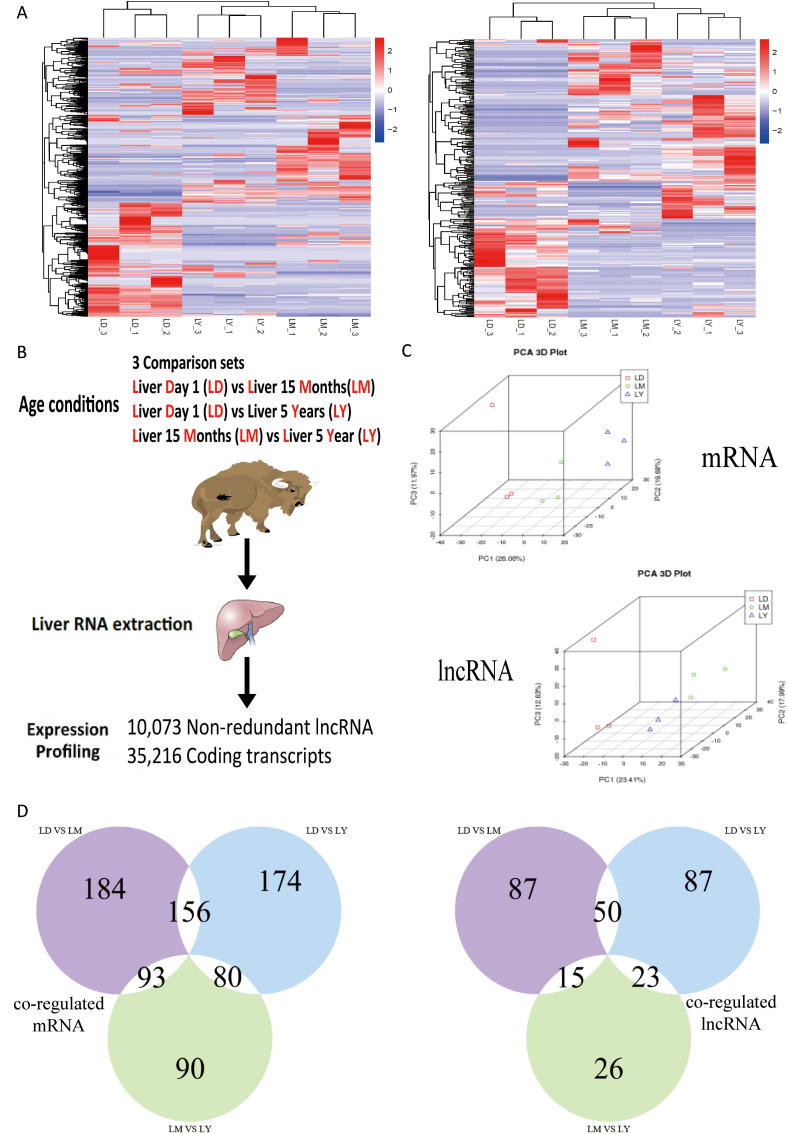
Expression profiles of lncRNA and mRNA in the liver at different age stages. **(A)** Hierarchical clustering and principal component analysis of differentially expressed mRNAs and lncRNAs in yak liver under different age conditions. Differentially expressed genes were identified as significantly different using one-way analysis of variance (ANOVA). **(B)** Experimental workflow. Each metabolic condition included three yaks, and the expression profiles of mRNAs or lncRNAs were analyzed in nine liver samples. **(C)** PCA analysis of differentially expressed mRNAs and lncRNAs in yak liver. **(D)** Distribution of the number of age-specific and co-regulated mRNAs and lncRNAs in the liver.

mRNA and lncRNA transcripts were detected in this study, and the current expression profile can be used to compare lncRNA and mRNA transcripts in yak liver at different developmental stages. Hierarchical cluster analysis was performed on all expressed transcripts, and the results showed that the mRNA expression profiles clearly divided all samples into three distinct groups classified by age stage, and these samples clustered closely together within each group at different developmental age stages (Fig 1A). However, what interests us is that the lncRNA expression results also showed almost identical sample clustering patterns, indicating that the lncRNA expression profile can serve as a signal similar to that of protein-coding mRNA (Fig 1A). Using RNA sequencing technology, 35,216 mRNAs and 10,073 lncRNAs were detected in transcripts from yak liver at three different developmental stages (Fig 1B). In addition, all samples were divided into multiple different groups by performing principal component analysis (PCA) on all regulated transcripts, including mRNA and lncRNA (Fig 1C), suggesting that regulated lncRNA and mRNA transcripts may act synergistically in related biological processes. Furthermore, to determine its functional connectivity, the lncRNA-mRNA network was analyzed by performing correlation analysis on the samples. By comparing LD with LM, 433 mRNAs and 152 lncRNAs were identified as being regulated; by comparing LD with LY, 410 mRNAs and 160 lncRNAs were identified as being regulated; and by comparing LM with LY, 263 mRNAs and 64 lncRNAs were identified as being regulated (Fig 1D), indicating that their expression levels are strictly regulated by yak age conditions.

### Functional analysis of differentially expressed genes (DEGs) in yak liver at different age stages

**Fig 2:**
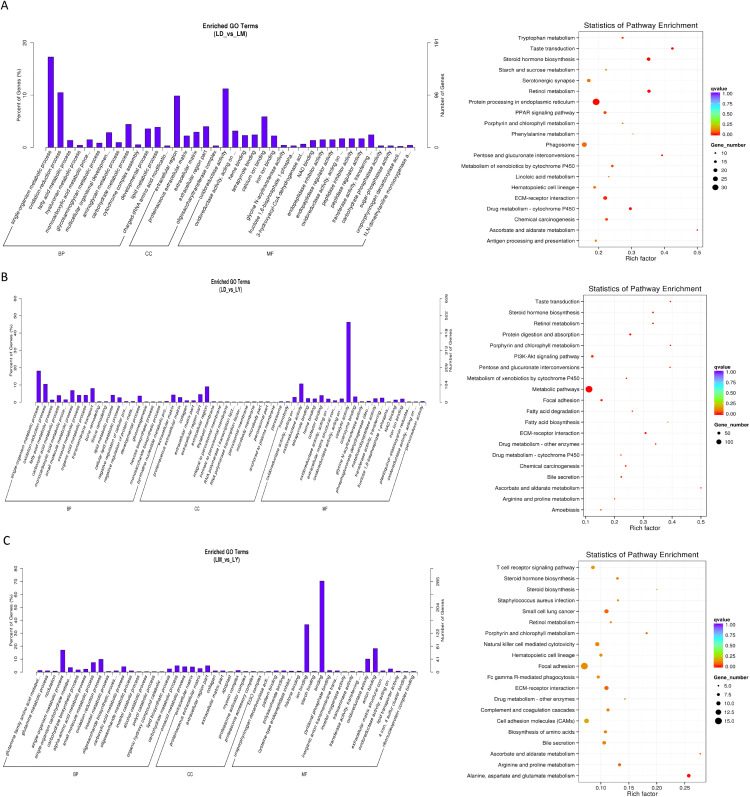
GO and KEGG pathways of differentially expressed mRNAs in beef liver at different age stages. (A) GO and KEGG pathways used to compare differentially expressed mRNAs between the LD and LM groups in yak liver. (B) GO and KEGG pathways used to compare differentially expressed mRNAs between the LD and LY groups in yak liver. (C) GO and KEGG pathways used to compare differentially expressed mRNAs between the LM and LY groups in yak liver.

In the comparison between LD and LM, DEGs were enriched in multiple GO funtional categories, including metabolism, ion binding, and developmental processes. In KEGG pathway analysis, DEGs were also enriched in metabolism, biosynthesis, extracellular matrix-receptor interactions, and other areas (Fig 2A). In the comparison between LD and LY, enriched GO functional categories included metabolism, tissue remodeling, and developmental processes. In KEGG pathway analysis, DEGs were also enriched with terms related to metabolism, biosynthesis, and PI3K-Akt signaling pathways (Fig 2B). Compared with LY, similar functional GO categories such as metabolism, biosynthesis, and redox processes were enriched in LM. In KEGG pathway analysis, DEGs were also enriched in terms related to metabolism, biosynthesis, and focal adhesion (Fig 2C). All these results indicate that metabolism-related functions develop during the developmental process of yaks.

### Predicting the function of differentially expressed lncRNAs through lncRNA and mRNA

**Fig 3:**
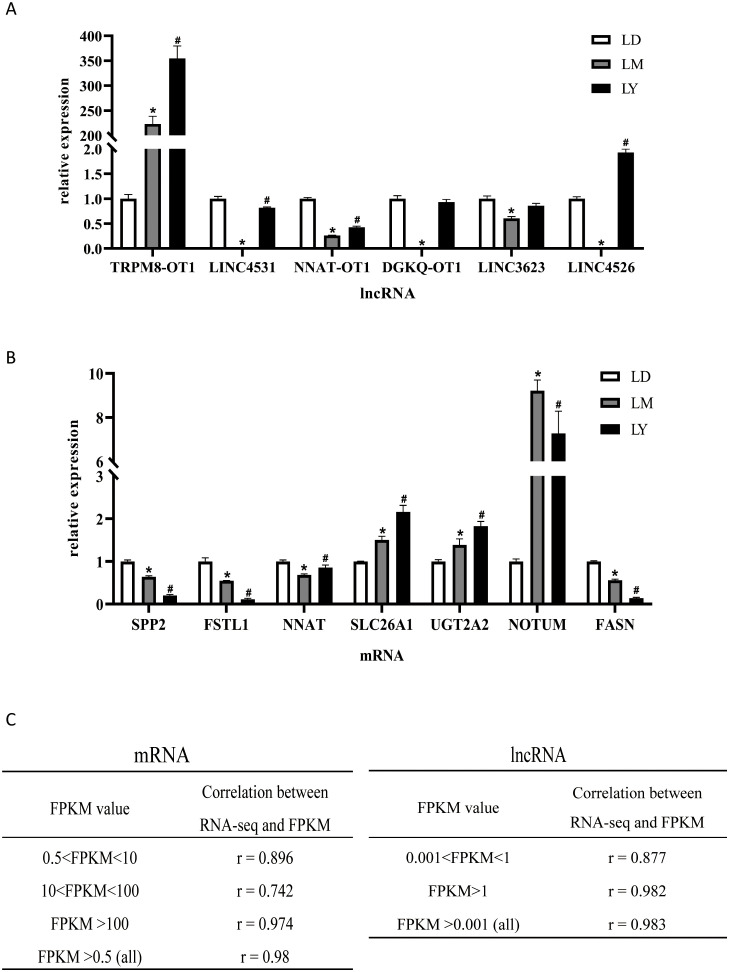
Functional prediction of LncRNA at different developmental stages based on LncRNA-mRNA co-expression correlation. **(A)** Differentially expressed LncRNA TCONS_00098792 in yak liver. **(B)** Differentially expressed LncRNA XLOC_045379 in yak liver. **(C)** Differentially expressed lncRNA XLOC_021536 in yak liver. **(D)** Differentially expressed lncRNA XLOC_041441 in yak liver. **(E)** Differentially expressed lncRNA TCONS_00032537 in yak liver. **(F)** Differentially expressed lncRNA XLOC_183608 in yak liver.

By analyzing the association between lncRNA and mRNA expression interaction networks, we predicted the functions of lncRNAs that are dynamically regulated at different developmental stages. From a total of 288 differentially expressed lncRNAs, we selected 88 lncRNAs that were regulated across at least two age comparisons. To examine the specific functions of these metabolism-related lncRNAs, we randomly picked six of them for further analysis. Functional analysis was performed using six lncRNAs as examples, and correlation analysis predicted their roles in metabolism or cell differentiation. TCONS_00098792 and XLOC_045379 were predicted to be associated with lipid metabolic processes (Fig 3A and B), while XLOC_021536 and XLOC_041441 were associated with collagen metabolism processes and protein metabolism processes in the liver, respectively (Fig 3C and D). TCONS_00032537 and XLOC_183608 were found to be enriched in cell proliferation and transport processes (Fig 3E and F). These results indicate that the established co-expression network can effectively predict the potential metabolic functions of age-regulated and metabolically sensitive lncRNAs.

**Fig 4:**
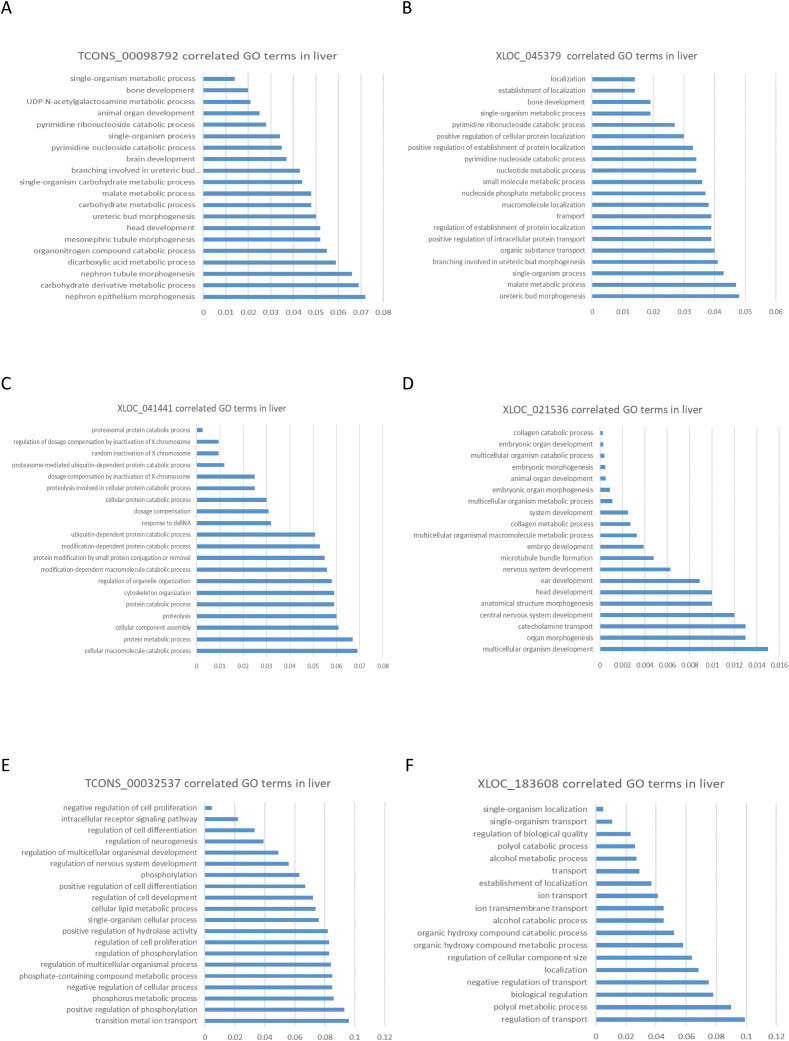
qRT-PCR validation of differentially expressed LncRNA and mRNA. (A) Validation of LncRNA RNA-seq results via quantitative real-time PCR analysis. The expression levels of six LncRNAs were measured. Error bars represent standard error of the mean (SEM). ^*^ **P* *< 0.05 (LD vs. LM), ^#^ **P* *< 0.05 (LD vs. LY). (B) Validation of mRNA RNA-seq results via quantitative real-time PCR analysis. The expression levels of 7 mRNAs were measured. Error bars represent SEM. ^*^ **P* *< 0.05 (LD vs. LM comparison), ^#^ **P* *< 0.05 (LD vs. LY comparison). (C) Pearson correlation between the log-transformed fold change of differentially expressed mRNAs and lncRNAs and RNA sequencing results.

These results indicate that the established co-expression network can effectively predict the potential metabolic functions of age-regulated, metabolism-sensitive lncRNAs in yak liver. The dynamic regulation of six randomly selected lncRNAs across different age comparisons was verified by qRT-PCR, confirming the feasibility of this method. Randomly selected six lncRNA GO functional enrichment analysis results showed significant differences between LM and LY compared with LD (Fig 4A and B). Pearson correlation analysis showed that RNA-seq and qRT-PCR detection of mRNA and lncRNA expression were highly consistent, verifying the reliability of the sequencing results (Fig 4C).

### Verify the intergroup expression differences of TCONS_00098792 and target genes

**Fig 5:**
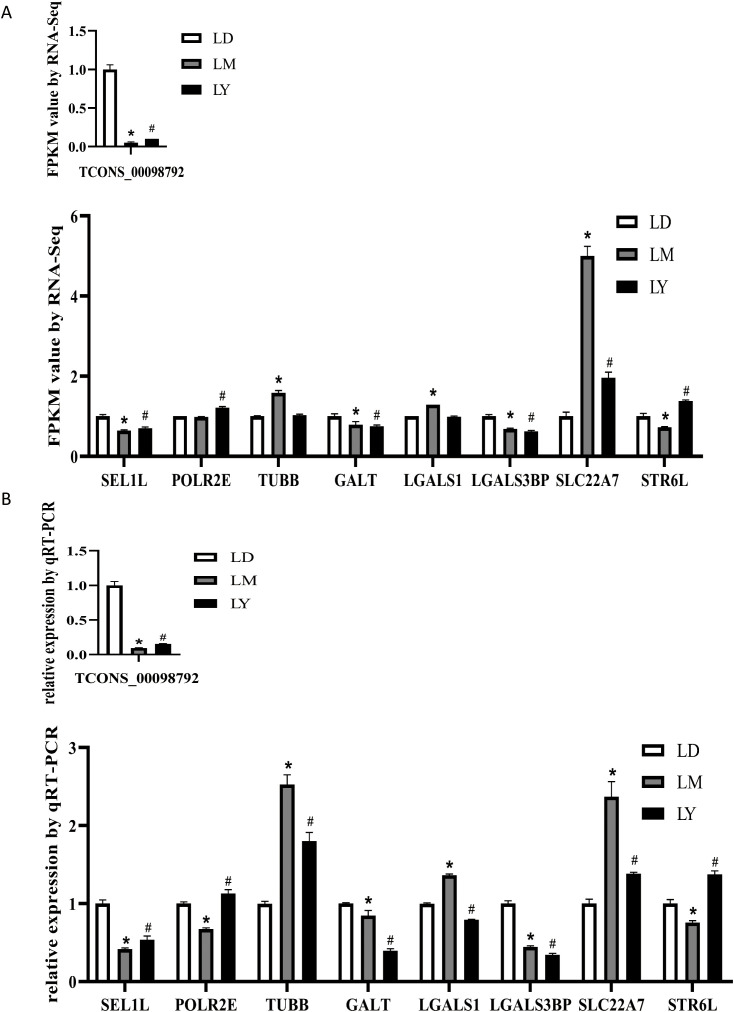
TCONS_00098792 and target cell expression levels. **(A)** RNA sequencing results of the expression levels of TCONS_00098792 and its target genes, * **P* *< 0.05 (LD and LM), ^#^ *P* < 0.05 (LD and LY). **(B)** qRT-PCR results of the expression levels of TCONS_00098792 and its target genes, * **P* *< 0.05 (LD and LM), ^#^ **P* *< 0.05 (LD and LY).

TCONS_00098792 was chosen for further validation. RNA-seq and qRT-PCR technologies were used to analyze and verify the expression of TCONS_00098792 and its target genes in the three age stages of LD, LM, and LY. The detection results showed that the expression level of TCONS_00098792 in LD was significantly higher than that in LM and LY (*P* < 0.05). The expression levels of the target genes of TCONS_00098792 also showed significant differences (*P* < 0.05) (Fig 5A and B).

## Discussion

Due to the difficulty in predicting the functions of lncRNAs, discovering and identifying lncRNAs that regulate metabolism has become a significant challenge. More importantly, lncRNAs are much less functionally conserved than mRNAs, even among closely related model organisms [32]. Most lncRNAs are unique to different animals, making it difficult to infer their functions through sequence alignment or evolutionary records [33]. Comparative genomics methods have been widely used to reveal the potential functions of novel protein-coding genes based on information from homologous genes or protein domains, but these methods have been proven ineffective in determining the functions of lncRNAs [34].

Metabolism is critical to almost all biological life activities, and all organisms typically regulate key metabolic pathways by rewiring metabolic flows. Therefore, rigorous functional measurements are needed to identify key points in metabolic regulation. Human organic anion transporter 2 (hOat2; encoded by the SLC22A7 gene) has been shown to possess functional diversity as a multi-substrate transporter, facilitating the transmembrane transport of cyclic guanosine monophosphate (cGMP), structurally diverse organic anions, and nicotinic acid. This transport activity makes hOat2 a key regulator of metabolic homeostasis, as these substrates play central roles in critical cellular processes such as nucleotide signaling, detoxification pathways, and nicotinamide adenine dinucleotide (NAD) biosynthesis, which collectively regulate the body’s metabolic network [35–37]. Identifying lncRNAs that regulate metabolism and revealing their functional characteristics remains a challenging task in animal studies. Currently, only a few lncRNAs have been reported to have a regulatory effect on metabolism [38–41]. The lncRNA LINC01056 plays a critical regulatory role in HCC (hepatocellular carcinoma) by suppressing PPARα-mediated FAO (fatty acid oxidation), thereby modulating sorafenib sensitivity [42]. These findings identify LINC01056 as a potential regulator of hepatic lipid metabolism and drug response pathways. However, there have been very few reports on the function of lncRNA in yak. It is worth noting that the value of elucidating the explicit lncRNA-mRNA regulatory network and its role in metabolic pathways for enhancing research understanding has also been validated in plant studies. In a study of the xylem of Populus euramericana ‘Zhonglin46’, high-throughput sequencing technology identified 14028 putative lncRNA transcripts, 4,525 differentially expressed lncRNAs (DELs), and 24,546 differentially expressed mRNAs (DEMs). DELs participate in pathways such as phenylpropanoid and lignin biosynthesis, as well as starch and sucrose metabolism, through cis- and trans-regulatory mechanisms [43].

For most lncRNAs, their functions have not been fully studied in depth. To further explore the function of lncRNAs in animal models, a critical first step is to accurately infer their functional roles. Investigating the involvement of lncRNAs in metabolic pathways across different developmental stages can help reveal uncharacterized regulatory mechanisms of complex metabolic processes. Specifically, to gain a deeper understanding of metabolic processes in the yak liver, identifying and characterizing more lncRNAs is essential. Establishing a workflow for lncRNA functional annotation will accelerate the screening and identification of metabolism-related lncRNAs, providing new insights into the study of complex metabolic networks.

Due to the non-coding nature of lncRNAs, it is not possible to directly determine their roles in metabolic processes. To overcome this difficulty, this study established a method for detecting functional lncRNAs through interaction analysis of lncRNAs in the livers of yaks of different ages. This integrated approach effectively narrowed down 288 age-related lncRNAs to 88 potential metabolism-related lncRNAs, which were differentially expressed across ages. Additionally, high-throughput RNA sequencing was intergrated to identify metabolism-related lncRNAs and established a workflow for discovering and characterizing functional lncRNAs in yaks of different ages. The data from this experiment support the idea that lncRNAs are key components of metabolic processes. Both lncRNA and mRNA expression profiles showed coordinated changes with different developmental stages. Moreover, the expression of metabolism-related lncRNAs in the liver is regulated by age, with their levels often changing significantly in yaks of different ages, supporting the potential biological significance of these lncRNAs. To verify whether this integrated transcriptomic analysis could reliably identify lncRNAs as metabolic regulators, the specific metabolic function of TCONS_00098792 was validated in the mouse liver AML12 cell line. These results indicated that TCONS_00098792 may potentially exert its metabolic regulatory role in yak liver by regulating SLC22A7, and this integrative analysis method also proves efficient for predicting metabolic lncRNAs, which could accelerate the identification of key lncRNA metabolic regulators. However, the results of this study are limited in terms of tissue specificity, as they focus only on the liver and do not involve other metabolism-related organs. At the same time, given the low functional conservation of lncRNAs among closely related model organisms, the cross-species applicability of the study conclusions may be limited.

## Conclusion

This study systematically analyzed the mRNA and lncRNA expression characteristics of yak liver at three developmental stages through integrated transcriptomic sequencing analysis. A total of 35,216 mRNAs and 10,073 lncRNAs were detected from the transcripts of yak liver at three different developmental stages. Through lncRNA-mRNA co-expression interaction network analysis, 88 metabolism-related lncRNAs were screened from 288 differentially expressed lncRNAs, and their potential functions in lipid metabolism, collagen remodeling, and protein transport were predicted. The age-dependent expression patterns of some lncRNAs were verified through qRT-PCR experiments, which initially revealed the role and importance of lncRNAs in yak liver metabolism.

## Supporting information

S1 File Supporting Information. (ZIP)
