# Supplementary figures and images for: Integrative transcriptomics analysis reveals the metabolic regulatory functions of lncRNA in the livers of yak at different age stages
Source: PLoS One. 2025 Oct 8;20(10):e0333944. doi: 10.1371/journal.pone.0333944 (PMC12507216; doi:10.1371/journal.pone.0333944)

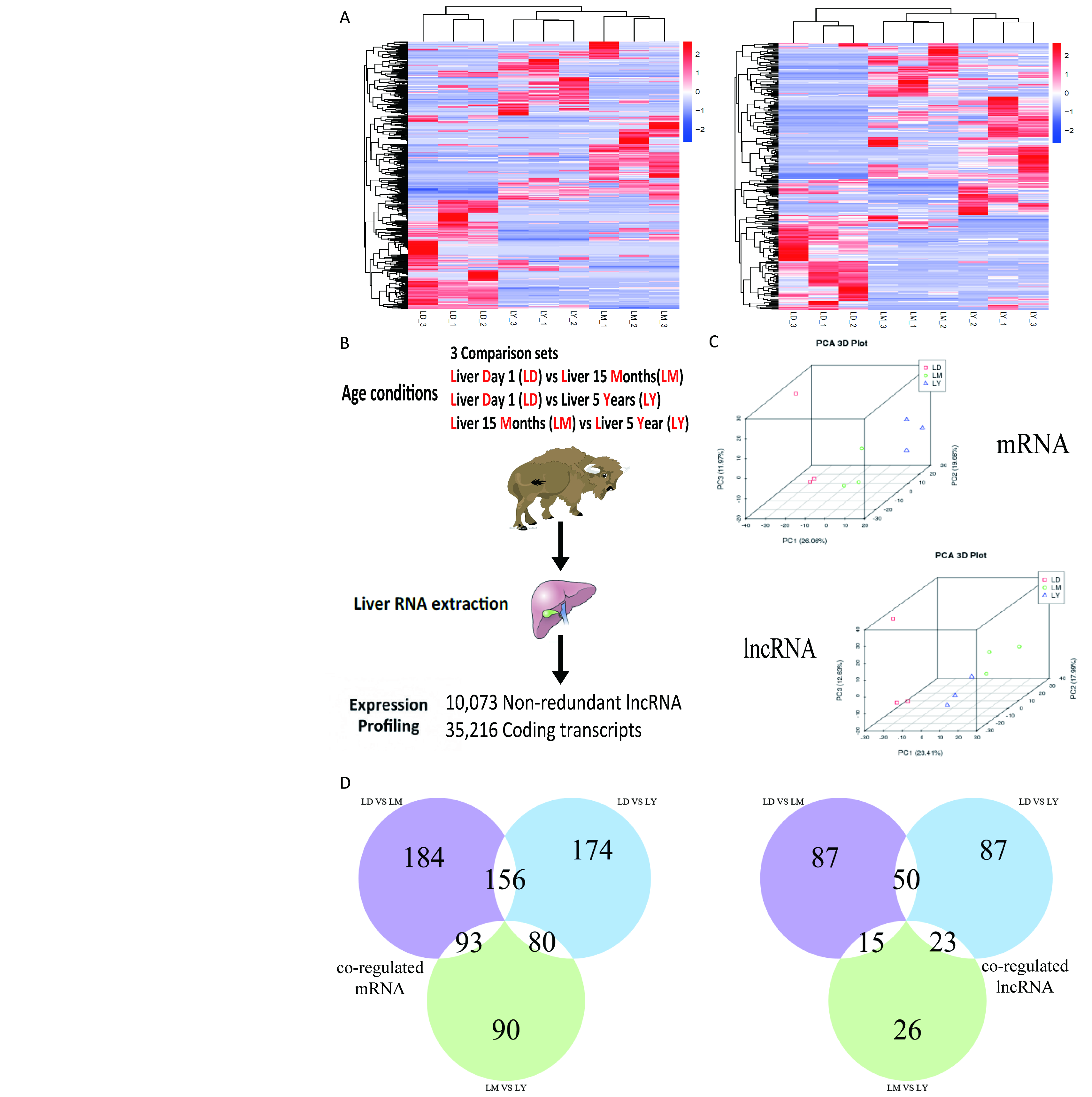

Supplement: S1 File — (ZIP) [file pone.0333944.s001.zip › Supporting Information/Supporting Information/lncRNA/Fig 1.tif]

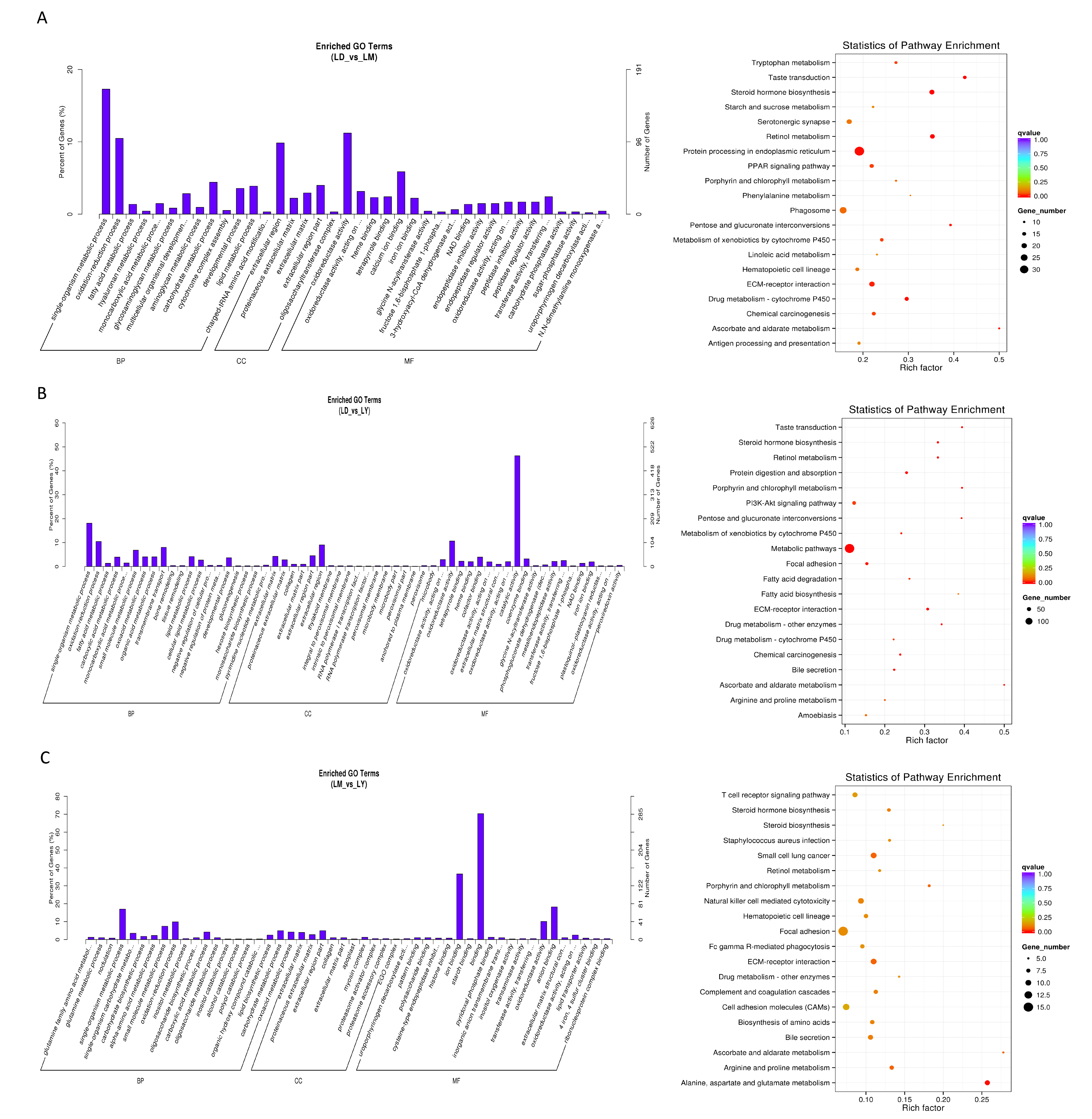

Supplement: S1 File — (ZIP) [file pone.0333944.s001.zip › Supporting Information/Supporting Information/lncRNA/Fig 2.tif]

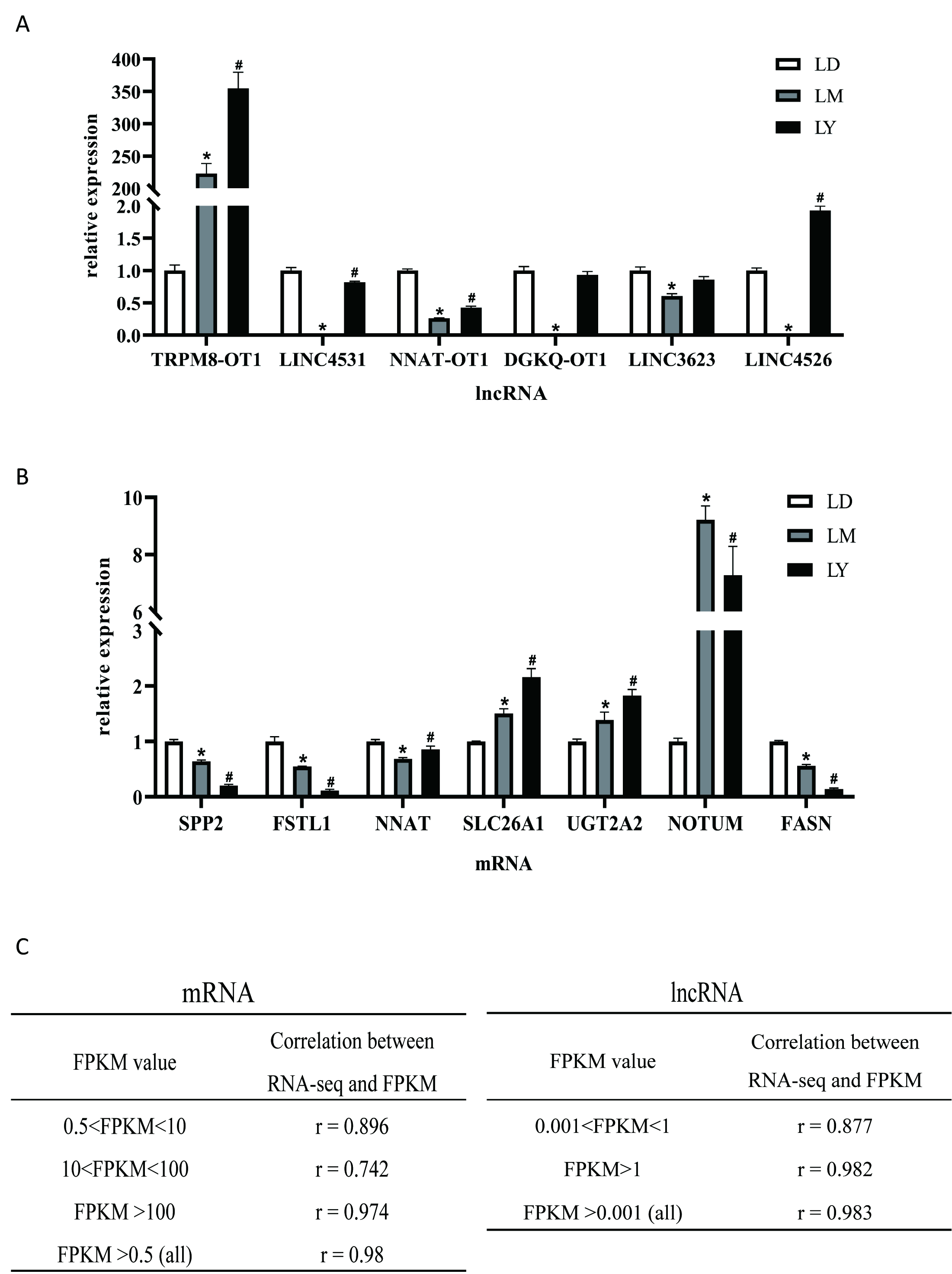

Supplement: S1 File — (ZIP) [file pone.0333944.s001.zip › Supporting Information/Supporting Information/lncRNA/Fig 3.tif]

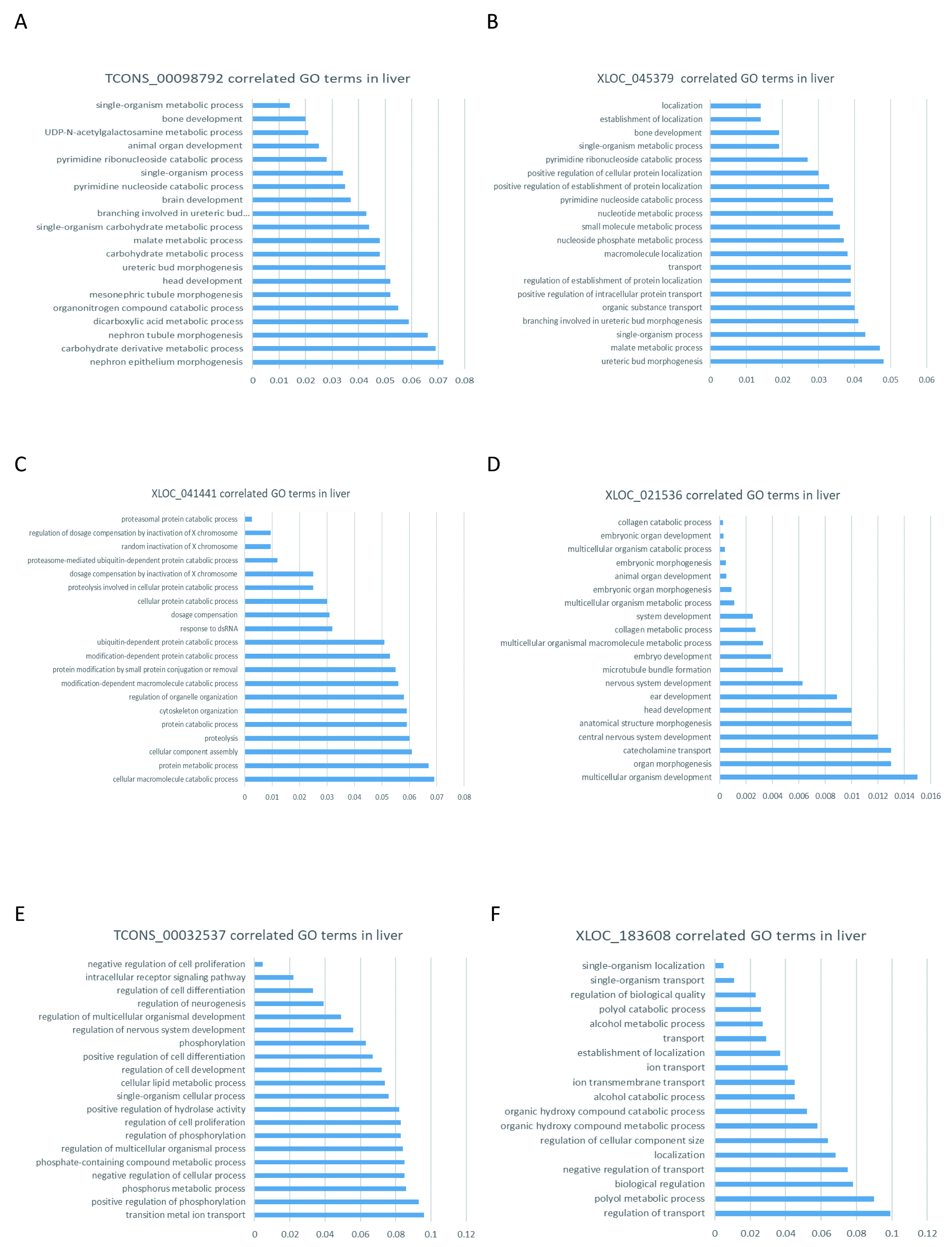

Supplement: S1 File — (ZIP) [file pone.0333944.s001.zip › Supporting Information/Supporting Information/lncRNA/Fig 4.tif]

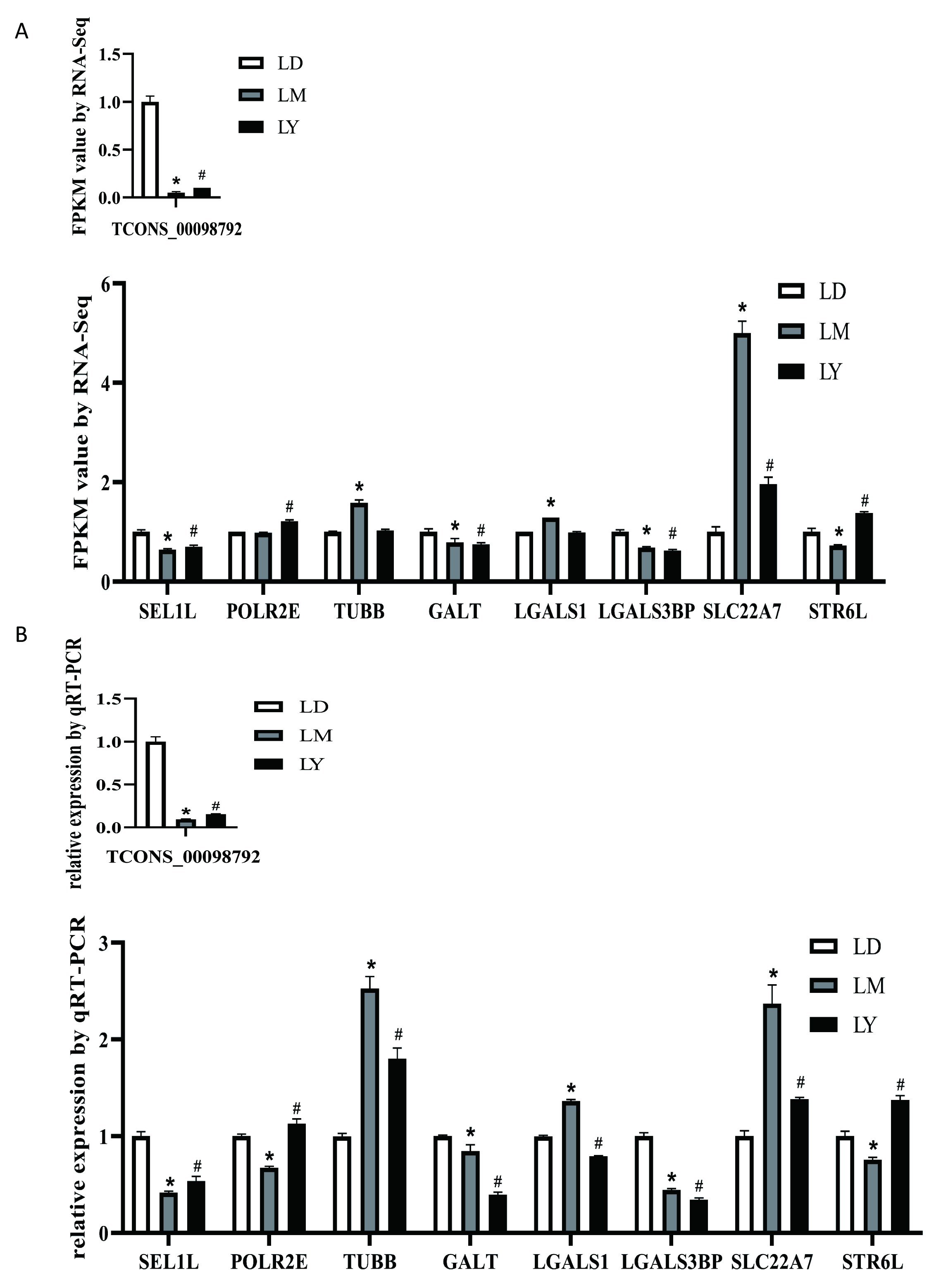

Supplement: S1 File — (ZIP) [file pone.0333944.s001.zip › Supporting Information/Supporting Information/lncRNA/Fig 5.tif]

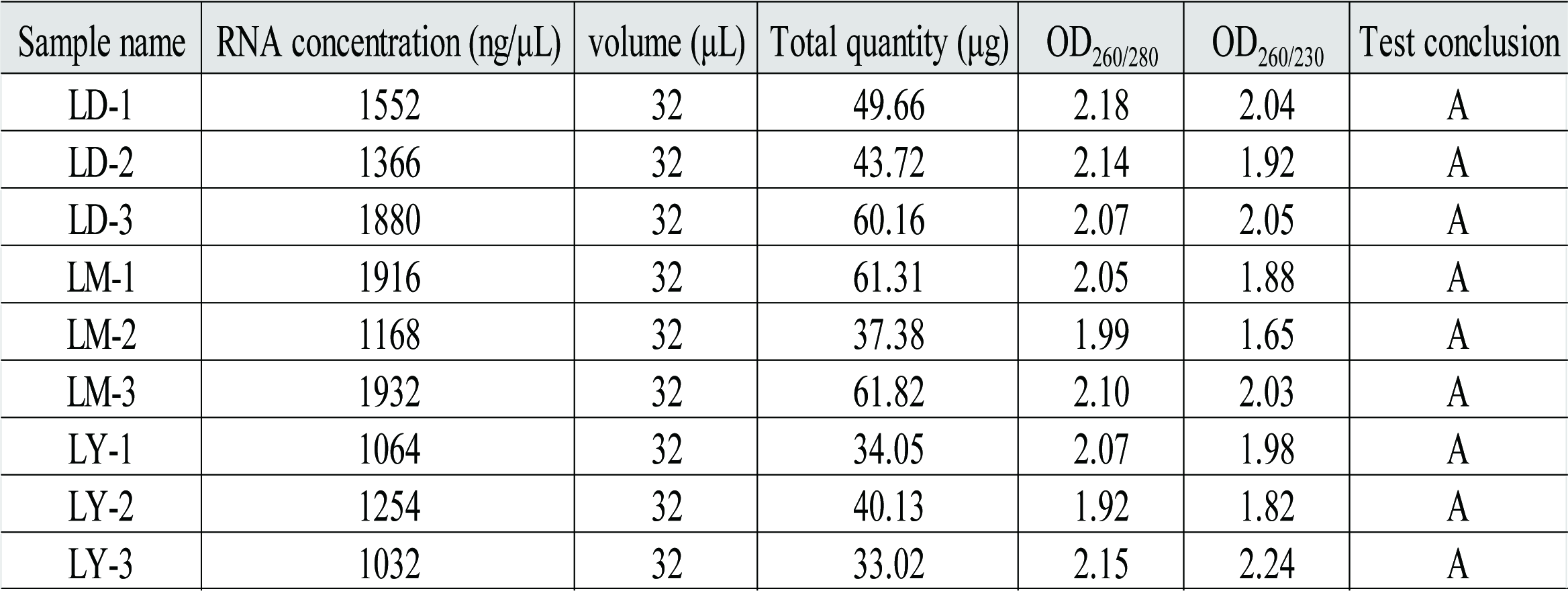

Supplement: S1 File — (ZIP) [file pone.0333944.s001.zip › Supporting Information/Supporting Information/lncRNA/Table 1.tif]

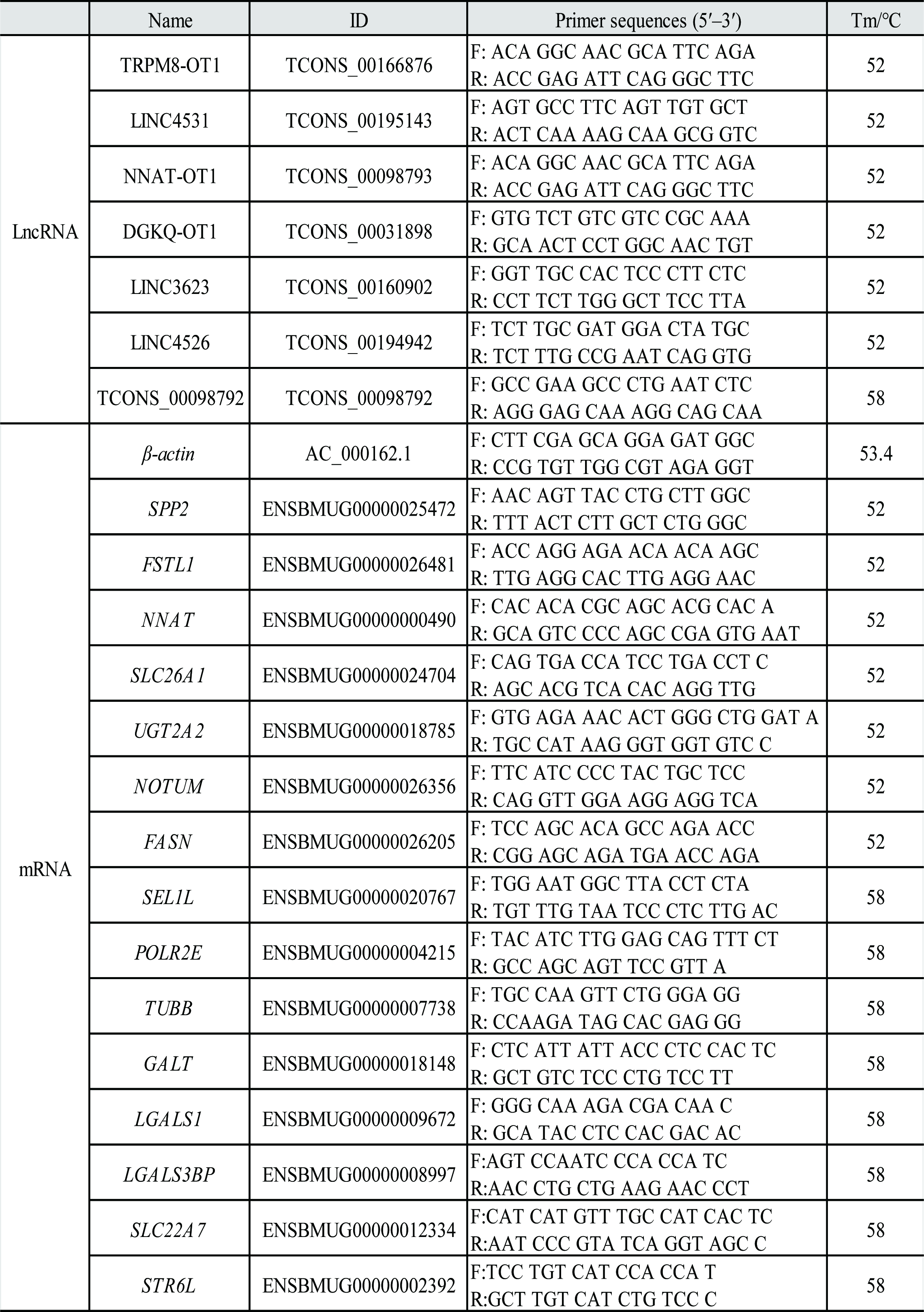

Supplement: S1 File — (ZIP) [file pone.0333944.s001.zip › Supporting Information/Supporting Information/lncRNA/Table 2.tif]
